# Doxorubicin chemotherapy affects the intracellular and interstitial free amino acid pools in skeletal muscle

**DOI:** 10.1371/journal.pone.0195330

**Published:** 2018-04-04

**Authors:** Sergio Fabris, David A. MacLean

**Affiliations:** 1 Biomolecular Sciences, Laurentian University, Sudbury, Ontario, Canada; 2 Division of Medical Sciences, Northern Ontario School of Medicine, Sudbury & Thunder Bay, Ontario, Canada; Institute of Subtropical Agriculture, Chinese Academy of Sciences, CHINA

## Abstract

Skeletal muscle (SM) health and integrity is dependent on the dynamic balance between protein synthesis and degradation, and central to this process is the availability of amino acids (AA) in the amino pool. While Doxorubicin (DOX) remains one of the most widely used chemotherapeutic agents for the treatment of solid and hematological malignancies, little is known of the effect of the drug on SM, particularly its effect on the availability of amino acids in the tissue. The purpose of this study was to examine the effect of DOX administration on vascular, interstitial and intracellular concentrations of AA in SM of the rat up to 8 days after the administration of a 1.5 or 4.5 mg/kg i.p. dose of DOX. In the plasma, total amino acids (TAA) were significantly increased compared to control where greater (P<0.05) concentrations were observed following the 1.5 mg/kg dose compared to the 4.5 mg/kg dose. Compared to control, the 1.5 mg/kg dose resulted in an increase (P<0.05) in interstitial TAA whereas the 4.5 mg/kg resulted in a sustained decrease (P<0.05). Intracellular TAA, essential amino acids (EAA) and branched-chain amino acids (BCAA) where significantly increased in each muscle group analyzed, following the 1.5 and 4.5 mg/kg doses compared to control. This study provides important insight into the amino acid response following DOX chemotherapy and presents a substantial foundation for future studies focused on reducing SM damage and recovery by targeting amino acid metabolism.

## Introduction

Skeletal muscle represents the largest organ in the human body, composing ~40% of the total body weight and in addition to its mechanical function, skeletal muscle plays a key role in whole body metabolism [1, 2]. Muscle mass is dependent on the dynamic balance between protein synthesis and degradation, and central to this process is the availability of amino acids in the free amino acid pool. In the human body, approximately 130 gms of free amino acids are present in the skeletal muscle intracellular space [3] and, in addition to its role in muscle protein turnover, amino acids participate in numerous metabolic reactions that take place in tissues throughout the body. Perturbation in the dynamic balance between protein synthesis and degradation where net protein breakdown is greater than that of synthesis in skeletal muscle can result in a cachectic, or muscle wasting, condition.

Cachexia is a serious, life-threatening condition associated with several pathologies including cancer [4–6]. Cancer cachexia affects 50–80% of cancer patients and accounts for approximately 20% of cancer-related deaths [7, 8]. Cachectic patients with greater than 15% weight loss exibit imparied physiological function and a drastic reduction in quality of life. Patient death normally occurs when weight loss exceeds 30% [8]. Additionally, cachectic patients are less tolerant to chemotherapy, radiation therapy and the response to these therapeutic strategies are significantly reduced [9, 10]. The use of anti-cancer chemotherapeutics such as that of Doxorubicin may play a role in the onset of cachexia in cancer patients undergoing chemotherapy.

Since its discovery, Doxorubicin (DOX) remains one of the most widely used chemotherapeutic agents for the treatment of various tumors [11]. DOX cytotoxicity has been attributed to DNA intercalation, inhibition of DNA topoisomerase II alpha (TOP2A) and the formation of reactive oxygen species [12–14]. The clinical use of DOX is constrained by a well-documented dose-dependent and cumulative cardiotoxic side effect which leads to the development of congestive heart failure where cardiotoxicity has been attributed to its primary circulating metabolite, Doxorubicinol (DOXol) [15, 16].

Recent research conducted in our laboratory has quantified the previously unreported sequestering of DOX and DOXol in skeletal muscle and described the relationship between the muscular, interstitial and vascular compartments following the administration of DOX [17]. In skeletal muscle, the interstitial space represents an important active compartment located between the vasculature and the tissue and it has been shown to play a functional role in the regulation and integration of various metabolic substances. The microdialysis technique provides a unique opportunity to quantify the availability of amino acids in this compartment *in vivo*. [18].

The importance of skeletal muscle health and integrity on the positive prognosis and quality of life of a cancer patient is well known however the effect of chemotherapy on skeletal muscle has been relatively overlooked. Therefore, the purpose of the present study was to examine the effects of DOX administration on the intramuscular, interstitial and vascular concentrations of amino acids in an effort to delineate the effects of DOX on protein turnover.

## Results

Since the direction of all the individual amino acids following the administration of DOX are similar, changes in the amino acid pool will be descrbied via changes in total amino acids. essential amino acids and branched-chain amino acids. The total amino acids (TAA) include Asp, Ser, Glu, Gly, His, Arg, Thr, Ala, Pro, Cys, Tyr, Val, Met, Lys, Iso, Leu and Phe. The essential amino acids (EAA) in the rat are His, Arg, Thr, Val, Met, Lys, Iso, Leu, Phe and branched-chain amino acids (BCAA) are Val, Iso and Leu. It must be noted that Phe could not be quantified in the intramuscular samples due to analytical limitations. Additionally, intramuscular Arg data has previously been published [19].

**Fig 1:**
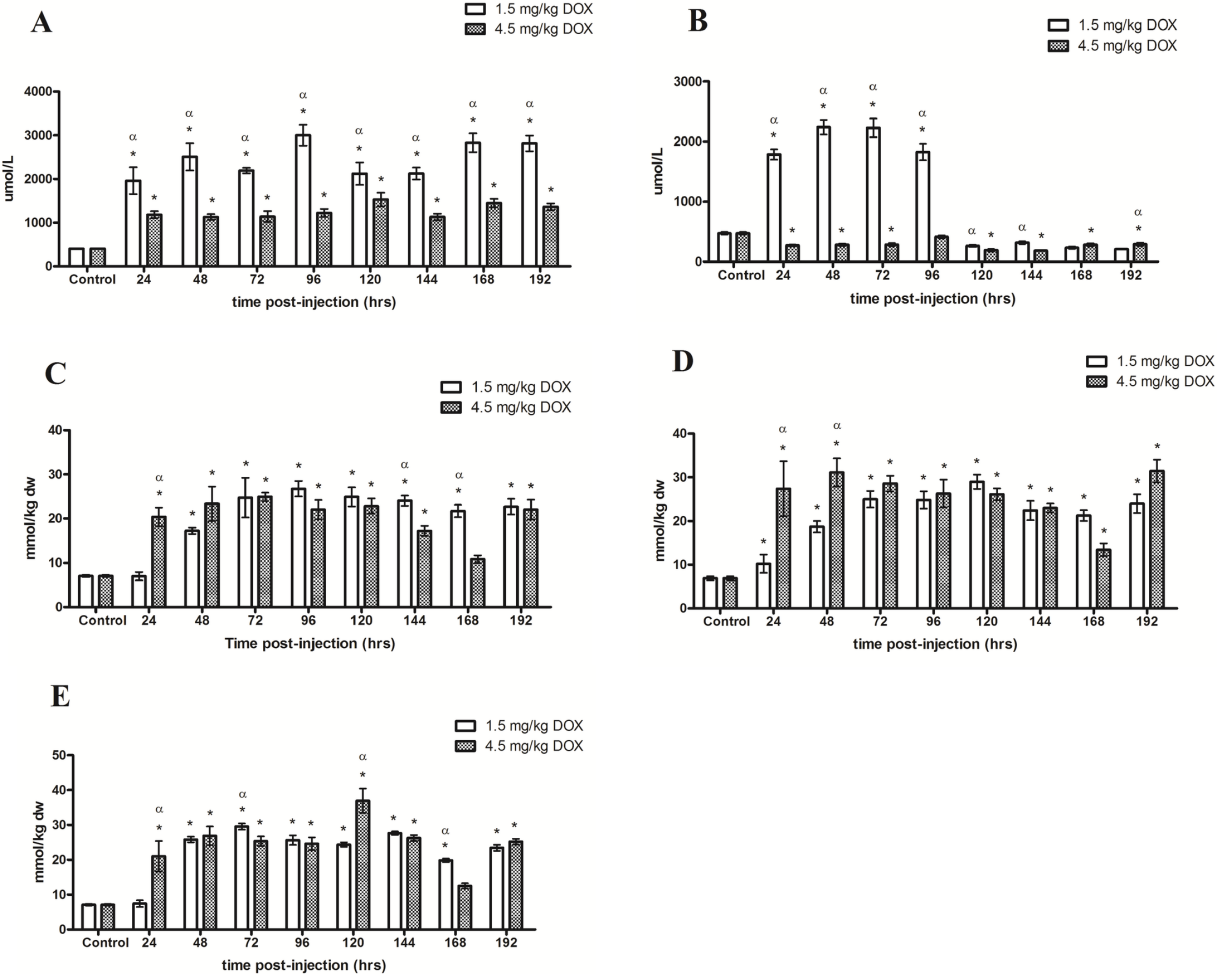
**Total amino acid concentrations in the (A) Plasma, (B) Dialysate, (C) White gastrocnemius, (D) Plantaris and (E) Soleus following the i.p. administration of 1.5 mg/kg DOX or 4.5 mg/kg DOX**. * Denotes significance compared to control. α Denotes difference (P<0.05) between administered doses.

The administration of the 1.5 and 4.5 mg/kg DOX resulted in a dose-related difference in arterial plasma amino acid concentrations (Table 1). Circulating arterial concentrations of TAA (Fig 1A) and EAA were increased (P<0.05) as a result of either administered dose compared to control however the increase in both the TAA and EAA were greater (P<0.05) following the 1.5 mg/kg dose compared to the 4.5 mg/kg dose. Circulating BCAA concentrations where significantly increased following the administration of both 1.5 and 4.5 mg/kg dose compared to control and were further elevated (P<0.05) at 96 hrs in the 4.5 mg/kg treatment group compared to the 1.5 mg/kg group. Although Ser, Cys, and Lys were increased (P<0.05) compared to control, their concentrations were not significantly different when compared between administered doses. Additionally, there were no changes in ammonia throughout the experiment following either administered dose.

**Table 1:** Arterial plasma amino acid concentrations.

Amino Acids	Dose	Timepoint								
	(mg/kg)	ctrl	24	48	72	96	120	144	168	192
Asp	1.5	13±1	175±31*†	230±44*†	195±10*†	298±24*†	195±27*	183±10*†	255±25*†	252±19*†
	4.5	13±1	103±5*†	102±3*†	100±10*†	135±23*†	138±14*	107±5*†	140±12*†	140±7*†
Ser	1.5	2.5±0.3	52±12*	39±7†*	23±3	30±4*	26±5*	24±3	25±3*	31±3†*
	4.5	2.5±0.3	37±1*	15±1†*	18±4*	25±4*	20±1*	15±3*	20±3*	21±1†*
Glu	1.5	0.05±0.01	194±42†	354±83*†	311±30*†	505±80*†	249±35*†	261±24*†	454±79*†	389±41*†
	4.5	0.05±0.01	65±10*†	94±23*†	81±13*†	68±6*†	143±30*†	77±8*†	119±12*†	76±7*†
Gly	1.5	12±1	135±24*	171±37†*	154±23†*	217±16†*	127±23*	94±4	155±16†*	133±12†*
	4.5	12±1	87±7*	64±12†	76±12†*	106±27†*	84±13*	75±10*	69±10†*	80±11†*
His	1.5	9±1	211±30*†	263±24*†	237±6*†	313±24*†	235±31*†	256±36*†	306±27*†	287±20*†
	4.5	9±1	100±21*†	38±7†	92±12*†	134±11*†	148±16*†	121±5*†	144±10*†	48±6†
Arg	1.5	9±01	60±13*	82±8*†	67±4*†	87±9*†	63±9*	72±14*	79±17*	96±4*†
	4.5	9±1	36±2*	34±3*†	36±5*†	36±3*†	47±6*	48±5*	45±4*	44±5*†
Thr	1.5	15±1	268±38*	306±28†*	308±14*†	381±34*†	300±41*†	354±51*†	384±15*†	369±26*†
	4.5	15±1	185±10*	161±15†*	137±15*†	182±30*†	182±17*†	163±15*†	182±9*†	189±7*†
Ala	1.5	44±3	262±40*†	320±29*†	247±8*†	340±29*†	242±34*	256±16*†	311±21*†	345±28*†
	4.5	44±3	152±12*†	139±5*†	149±12*†	156±12*†	200±22*	171±8*†	189±12*†	223±19*†
Pro	1.5	9±1	102±16*†	109±11*†	98±5*†	131±13*†	88±11*	91±5*†	120±12*†	129±9*†
	4.5	9±1	58±3*†	51±3*†	50±5*†	54±4*†	65±6*	59±7*†	67±4*†	73±3*†
Cys	1.5	193±5	8±1*	14±1*	18±3*	8±2*	14±3*	15±3*	20±3†*	14±2*
	4.5	193±5	10±1*	14±2*	12±2*	9±3*	13±3*	10±1*	10±1†*	13±1*
Tyr	1.5	2±0.1	38±5*†	51±7*†	43±4*†	49±3*†	34±3*	45±9*†	49±6*†	56±4*†
	4.5	2±0.1	24±2*†	23±3*†	21±3*†	20±2*†	23±4*	17±2*†	23±2*†	22±1*†
Val	1.5	14±1	107±16*	133±20*	122±16*	174±25*†	115±17*	151±23*†	172±16*†	179±10*†
	4.5	14±1	76±5*	98±11*	79±13*	80±14*†	97±8*	71±4*†	103±5*†	104±4*†
Met	1.5	1.4±0.1	36±5*†	41±6*†	36±2*†	44±5*†	34±5*	37±5†*	45±4*†	46±2*†
	4.5	1.4±0.1	24±0.5*†	22±2*†	20±2*†	19±2*†	23±2*	18±2†*	23±1*†	23±1*†
Lys	1.5	33±2	127±14*	155±13*	152±10*	197±26†*	172±14*	180±21*	207±24*†	187±7*†
	4.5	33±2	102±11*	123±10*	124±17*	114±13†*	164±15*	143±7*	131±12*†	135±9*†
Iso	1.5	6.4±0.4	45±7	65±11*	60±10*	87±14†*	63±8*	79±15*†	91±10*†	93±7*†
	4.5	6.4±0.4	32±2*	50±6*	39±7*	37±7†*	49±5*	35±2*†	52±3*†	53±2*†
Leu	1.5	11±1	77±12*	109±20*	101±16*	142±22*†	106±14*	131±24*†	153±17*†	155±12*†
	4.5	11±1	56±4*	83±9*	67±13*	65±13*†	84±7*	60±4*†	86±4*†	87±5*†
Phe	1.5	1.4±0.1	32±4*†	35±3*†	35±2*†	42±4*†	32±4*†	35±4*†	44±4*†	43±2*†
	4.5	1.4±0.1	22±1*†	19±2*†	16±1*†	16±1*†	19±2*†	15±1*†	21±2*†	20±0.3*†
NH_3_	1.5	33±1	34±8	31±4	26±2	38±6	25±2†	23±2	28±1	27±3
	4.5	33±1	30±3	25±2	24±2	26±2	32±2†	26±1	30±3	31±3
EAA	1.5	95±5	930±131*†	1154±107*†	1084±52*†	1346±108*†	1088±120*	1249±175*†	1372±75*†	1396±44*†
	4.5	95±5	605±40*†	608±52*†	593±77*†	660±80*†	795±72*	611±38*†	765±44*†	667±46*†
BCAA	1.5	31±2	229.6±34*†	307±51*	284±41*†	402±61*†	283±35*	361±62*†	416±42*†	427±29*†
	4.5	31±2	164±11*†	231±26*	185±33*†	181±34*†	231±20*	139±29*†	240±12*†	244±11*†

All values are means ± SEM. Concentrations represented in μM concentrations.

* denotes significance compared to control.

† Denotes significance between doses.

The administration of DOX resulted in a dose-related difference in interstitial amino acids (Table 2). The 1.5 mg/kg dose resulted in a transient increase (P<0.05) in TAA (Fig 1B), EAA and BCAA as well as ammonia from 24–96 hrs compared to control. However Cys, Lys and Leu decreased (P<0.05) to concentrations lower than control after 96 hrs. TAA, EAA, BCAA and ammonia decreased (P<0.05) 24–192 hours following the administration of 4.5 mg/kg DOX as compared to control. Ser and Glu concentrations were increased (P<0.05) compared to control and Met was not significantly affected.

**Table 2:** Interstitial amino acid concentrations.

Amino Acids	Dose	Timepoint								
	(mg/kg)	ctrl	24	48	72	96	120	144	168	192
Asp	1.5	33±2	162±9*†	202±16*†	202±13*†	219±16*†	18±1†	20±1†	18±2	16±1
	4.5	33±2	18±1*†	19±1*†	23±2*†	39±2†	11±1*†	12±1*†	18±1*	18±2*
Ser	1.5	13±1	73±5*†	59±6*†	60±6*†	75±9*†	2±0.2†	2±0.2†	1±0.1†	2±0.2†
	4.5	13±1	3±0.4*†	7±1*†	7±1*†	6±1*†	21±3*†	13±1†	14±2†	10±1†
Glu	*1*.*5*	2±0	58±4*†	66±5*†	55±7*†	30±3*	21±2*†	22±2*†	16±2†	13±1
	*4*.*5*	2±0	12±1*†	16±1*†	22±2*†	39±4*	10±1*†	10±1*†	11±1*†	16±2*
Gly	1.5	46±3	145±8*†	152±11*†	133±9*†	132±9*†	24±1†	26±2†	22±2†	19±1†
	4.5	46±3	20±1*†	25±2*†	23±2*†	35±2*†	15±2*†	16±1*†	30±2*†	31±3*†
His	1.5	36±2	139±9*†	161±10*†	133±9*†	132±9*†	28±2†	30±2†	22±2†	19±1†
	4.5	36±2	20±1*†	25±2*†	23±2*†	35±2†	20±2*†	20±1*†	30±2†	30±3†
Arg	1.5	47±3	147±7*†	171±10*†	202±15*†	156±13*†	54±3†	63±5†	43±3†	36±3
	4.5	47±3	44±2†	44±4†	47±6†	61±4†	29±3*†	31±2*†	32±4†	34±4
Thr	1.5	29±2	120±13*†	171±10*†	38±5†	19±3†	22±2†	25±2†	14±4	20±2
	4.5	29±2	4±1*†	3±0.5*†	11±1*†	7±1*†	14±2*†	8±1*†	22±3	21±2
Ala	1.5	64±4	332±19*†	444±31*†	454±42*†	398±34*†	31±2†	42±3†	29±3†	27±2†
	4.5	64±4	37±3*†	40±3*†	36±3*†	67±5†	17±2*†	24±2*†	35±3*†	47±7*†
Pro	1.5	24±1	71±4*†	80±5*†	89±7*†	82±7*†	8±0.5†	11±1†	9±1	9±1
	4.5	24±1	10±1*†	12±1*†	10±1*†	17±1*†	5±1*†	6±0.4*†	9±1*	10±1*
Cys	1.5	8±1	24±1*†	27±2*†	28±2*†	20±2*†	3±0.1*†	3±0.2*†	2±0.2*†	2±0.1*†
	4.5	8±1	3±0.2*†	3±0.2*†	3±0.4*†	4±0.4*†	2±0.3*†	2±0.1*†	5±0.4*†	5±0.5*†
Tyr	1.5	4±0	16±1*†	15±1*†	14±1*†	13±1*†	2±0.1†	3±0.2†	2±0.2	2±0.1
	4.5	4±0	3±0.1*†	3±0.3*†	3±0.2*†	4±0.3†	1±0.2*†	2±0.1*†	2±0.3*	2±0.2*
Val	1.5	27±1	75±3*†	103±8*†	126±10*†	102±11*†	11±1†	14±1†	12±1	11±1
	4.5	27±1	22±1†	17±1*†	16±2*†	19±1*†	8±1*†	8±0.4*†	12±1*	12±1*
Met	1.5	3±0	21±1*†	20±1*†	21±2*†	19±2*†	3±0.1†	3±0.2†	2±0.2	2±0.1
	4.5	3±0	3±0.2†	3±0.2†	3±0.3†	4±0.3†	1±0.2*†	1±0.1*†	3±0.2	2±0.2
Lys	1.5	66±2	158±9*†	227±14*†	273±24*†	222±18*†	15±1*†	16±1†	16±2	12±1*†
	4.5	66±2	24±2*†	19±2*†	222±2*†	30±2*†	12±1*†	13±1*†	17±1*	17±2*†
Iso	1.5	11±1	25±1*†	43±4*†	52±5*†	37±5*†	5±0.3†	6±1†	6±0.5	5±0.3
	4.5	11±1	10±1†	6±0.4*†	7±1*†	8±1*†	3±0.5*†	4±0.2*†	6±1*	6±1*
Leu	1.5	19±1	51±2*	79±7*	94±8*	66±7*†	9±0.5*	11±1*†	10±1*†	9±1*†
	4.5	19±1	17±1*	11±1*	12±2*	14±1*†	6±1*	6±0.3*†	12±1*†	10±1*†
Phe	1.5	3±0	11±1*†	9±0.5*†	10±1*†	8±1*†	3±0.2†	3±0.2†	2±0.2	2±0.1
	4.5	3±0	2±0.2*†	3±0.2†	3±0.2*†	3±0.2†	2±0.2*†	1±0.1*†	2±0.2*	2±0.2*
NH_3_	1.5	36±3	156±9*†	215±11*†	254±17*†	107±8*†	22±1†	22±1†	16±1†	13±1†
	4.5	36±3	22±4*†	26±1*†	19±1*†	26±1*†	15±1*†	15±1*†	28±1*†	23±2*†
EAA	1.5	195±8	601±35*†	812±45*†	738±55*†	593±47*†	87±6†	106±8†	75±8	72±4†
	4.5	195±8	100±5*†	87±6*†	93±8*†	117±6*†	65±8*†	56±4*†	100±9*	98±10*†
BCAA	1.5	57±3	151±7*†	225±18*†	272±23*†	204±24*†	24±1†	40±3†	27±3	25±1
	4.5	57±3	48±3†	34±2*†	35±5*†	40±3*†	17±2*†	17±1*†	31±3*	28±3*

All values are means ± SEM. Concentrations represented in μM concentrations.

* denotes significance compared to control.

† Denotes significance between doses.

**Fig 2:**
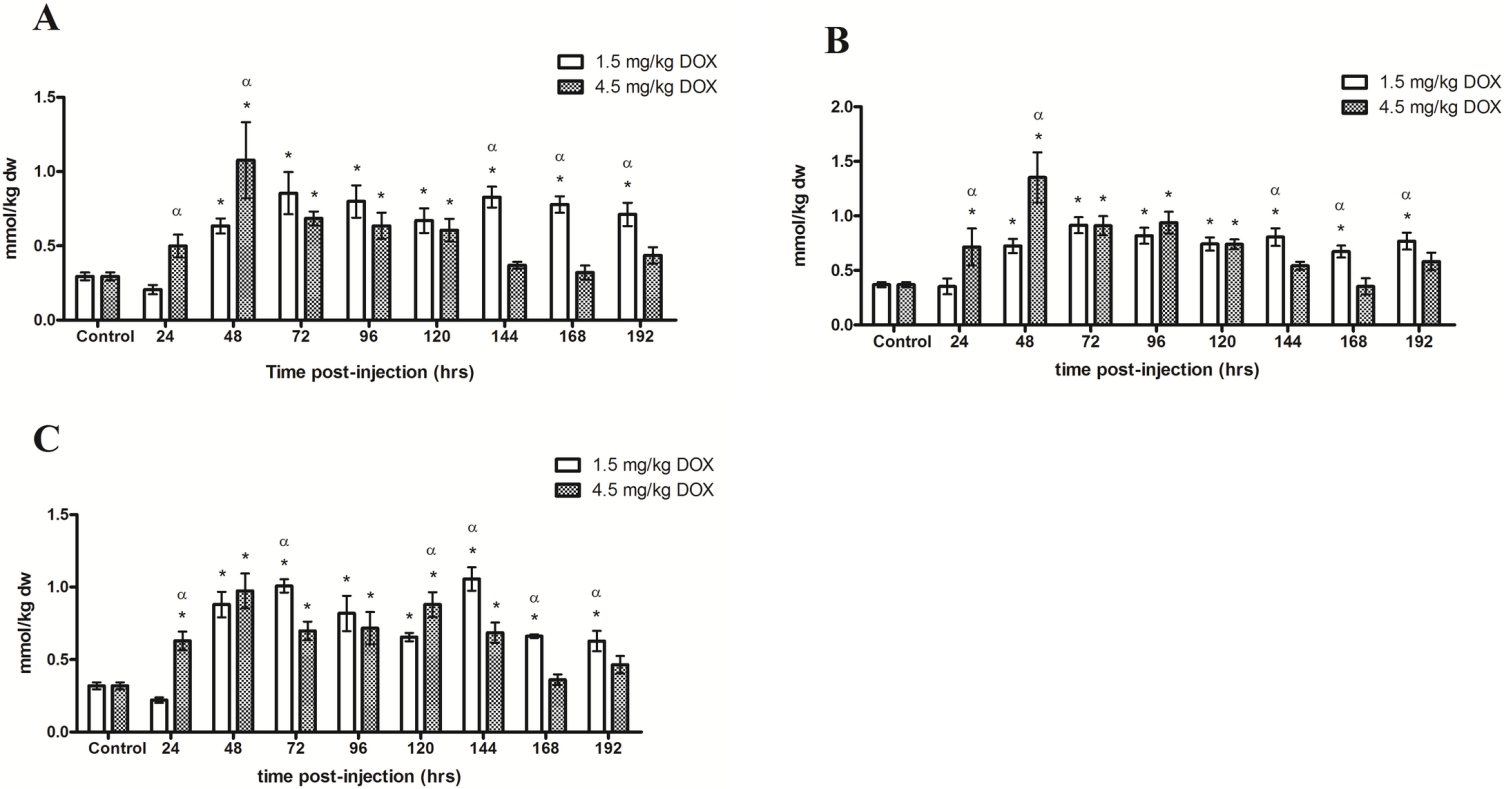
**Total intracellular concentrations of branched-chain amino acids (Val, Iso and Leu) in the (A) White gastrocnemius, (B) Plantaris and (C) Soleus following the i.p. administration of 1.5 mg/kg DOX or 4.5 mg/kg DOX**. * Denotes significance compared to control. α Denotes difference (P<0.05) between administered doses.

In the WG (Table 3), the administration of 1.5 and 4.5 mg/kg DOX increased (P<0.05) TAA (Fig 1C) and EAA concentrations. BCAA were significantly increased from 48–192 hrs following the 1.5 mg/kg dose whereas the 4.5 mg/kg dose increased (P<0.05) BCAA 48–120 hrs compared to control (Fig 2A). Following the 1.5 mg/kg dose, Cys significantly decreased compared to control and was undetectable after 72 hrs. Concentrations of Asp, Ser, Ala, Pro, Iso and Leu where greater after the 1.5 mg/kg dose compared to the 4.5 mg/kg dose and ammonia was unaffected in both treatment groups.

**Table 3:** Intracellular Amino Acids in the medial gastrocnemius.

Amino Acids	Dose	Time post-injection (in hours)								
	(mg/kg)	ctrl	24	48	72	96	120	144	168	192
Asp	1.5	0.19±0.01	0.28±0.05	0.63±0.09*	0.78±0.2*	0.99±0.05*†	0.67±0.1*†	0.71±0.1*†	0.64±0.08*†	0.53±0.05
	4.5	0.19±0.01	0.43±0.07	0.64±0.13*	0.65±0.06*	0.71±0.07*†	0.68±0.09*†	0.38±0.03†	0.28±0.04†	0.4±0.05
Ser	1.5	0.35±0.04	0.33±0.05	0.82±0.18	0.73±0.07	0.69±0.04	0.55±0.10	1.16±0.35*†	0.44±0.03†	0.86±0.18
	4.5	0.35±0.04	0.56±0.11	0.77±0.21*	0.53±0.05	0.57±0.05	0.37±0.09	0.29±0.05†	0.29±0.04†	0.57±0.08
Glu	1.5	0.03±0.01	0.15±0.02†	0.27±0.04*	0.45±0.10*	0.47±0.04*	0.46±0.03*	0.47±0.08*	0.38±0.05*†	0.37±0.05*
	4.5	0.03±0.01	0.32±0.05†	0.29±0.05	0.47±0.07*	0.55±0.1*	0.86±0.19*	0.43±0.05*	0.21±0.02†	0.45±0.04*
Gly	1.5	0.68±0.04	0.47±0.11†	1.65±0.41	2.22±0.62*	2.96±0.15*†	1.87±0.3*	1.8±0.29	1.63±0.24†	1.82±0.18
	4.5	0.68±0.04	1.66±0.3*†	1.64±0.31*	2.07±0.23*	2.23±0.24*†	2.07±0.25*	1.24±0.08	0.52±0.06†	1.8±0.22*
His	1.5	0.21±0.01	0.48±0.08†	1.28±0.26*	1.19±0.20*	1.56±0.12*	1.44±0.29*	1.48±0.27*	1.11±0.17*†	1.0±0.98*†
	4.5	0.21±0.01	0.79±0.1†	1.69±0.63*	1.17±0.1	1.4±0.17*	1.43±0.23*	0.93±0.05	0.61±0.12†	1.53±0.19*†
Thr	1.5	0.92±0.02	1.03±0.17†	2.76±0.63	3.82±0.57*	3.84±0.18*	5.33±0.95*	4.40±0.47*	4.11±0.64*†	4.98±0.49*
	4.5	0.92±0.02	3.35±0.32*†	4.65±0.93*	4.59±0.92*	3.79±0.46*	5.73±0.5*	4.08±0.47*	2.16±0.31†	4.59±0.57*
Ala	1.5	0.72±0.06	0.9±0.12†	1.6±0.16	2.08±0.57*	2.54±0.19*†	1.28±0.19	1.54±0.14†	1.28±0.17†	1.42±0.21
	4.5	0.72±0.06	1.81±0.29*†	2.2±0.51*	1.65±0.34	1.47±0.12†	1.49±0.22	0.76±0.07†	0.65±0.1†	1.06±0.07
Pro	1.5	0.11±0.01	0.08±0.01†	0.18±0.02	0.25±0.06*	0.27±0.02*	0.2±0.01	0.23±0.02*†	0.2±0.02†	0.18±0.02†
	4.5	0.11±0.01	0.18±0.03†	0.23±0.05*	0.26±0.01*	0.22±0.03*	0.22±0.04*	0.11±0.002†	0.08±0.01†	0.11±0.02†
Cys	1.5	0.47±0.02	0.02±0.001*†	0.04±0.004*†	n/d	n/d	n/d	n/d	n/d	n/d
	4.5	0.47±0.02	0.92±0.14*†	0.67±0.08†	0.38±0.09	n/d	n/d	n/d	n/d	n/d
Tyr	1.5	0.03±0.002	n/d	0.06±0.01†	0.11±0.03*	0.11±0.01*	0.09±0.003*	0.09±0.01*†	0.07±0.01*†	0.09±0.01*
	4.5	0.03±0.002	n/d	0.17±0.04*†	0.08±0.01	0.1±0.02	0.14±0.02*	0.06±0.01†	0.05±0.01†	0.1±0.01
Val	1.5	0.12±0.01	0.09±0.01†	0.26±0.03*	0.34±0.06*	0.33±0.05*	0.28±0.03*	0.34±0.03*†	0.31±0.03*†	0.29±0.03*
	4.5	0.12±0.01	0.2±0.03†	0.42±0.1*	0.29±0.02*	0.26±0.03	0.29±0.02*	0.16±0.01†	0.13±0.02†	0.2±0.03
Met	1.5	0.03±0.002	0.04±0.001†	0.07±0.01†	0.11±0.03*	0.1±0.01*	0.08±0.01*†	0.09±0.002*†	0.08±0.01*†	0.08±0.01*†
	4.5	0.03±0.002	0.07±0.01†	0.2±0.04*†	0.1±0.01*	0.1±0.02*	0.11±0.01*†	0.05±0.003†	0.04±0.01†	0.05±0.01†
Lys	1.5	0.42±0.04	0.27±0.03†	1.39±0.21*	0.94±0.42	1.51±0.21*	1.06±0.08	1.25±0.1*†	1.32±0.15*†	0.8±0.13
	4.5	0.42±0.04	0.72±0.13†	1.01±0.3	2.22±0.38*	1.21±0.26*	1.0±0.21	0.43±0.04†	0.35±0.01†	0.85±0.09
Iso	1.5	0.06±0.01	0.05±0.01†	0.14±0.01*	0.18±0.03*	0.16±0.03*	0.14±0.02*	0.18±0.02*†	0.16±0.01*†	0.15±0.02*†
	4.5	0.06±0.01	0.1±0.02†	0.24±0.06*	0.15±0.01	0.14±0.02	0.17±0.02*	0.08±0.01†	0.07±0.01†	0.08±0.01†
Leu	1.5	0.14±0.01	0.09±0.01†	0.24±0.02	0.33±0.06*	0.3±0.04*	0.26±0.04	0.31±0.03*†	0.3±0.02*†	0.27±0.03†
	4.5	0.14±0.01	0.2±0.03†	0.42±0.1*	0.25±0.02	0.23±0.04	0.23±0.02	0.13±0.01†	0.12±0.02†	0.16±0.03†
NH_3_	1.5	1.29±0.11	0.35±0.02†	0.71±0.1	2.03±0.78	2.23±0.34†	1.42±0.23†	1.35±0.15†	1.55±0.23†	0.98±0.16
	4.5	1.29±0.11	1.41±0.19†	1.26±0.26	1.21±0.06	0.86±0.09†	0.77±0.09†	0.61±0.04*†	0.83±0.11†	1.19±0.18
EAA	1.5	3.14±0.15	4.58±0.8†	11.5±1.4*	16.1±2.3*	17.1±1.0*	18.6±1.6*	16.7±1.2*	15.6±1.1*†	16.4±1.2*
	4.5	3.14±0.15	13.1±1.4*†	15.7±2.4*	17.8±0.5*	15.3±1.7*	16.2±1.1*	13.4±1.0*	8.0±0.6†	16.5±1.9*

All values are means ± SEM. Concentrations represented in mmol/kg dry weight.

* denotes significance compared to control.

† Denotes significance between doses. n/d denotes not detected.

TAA (Fig 1D) and EAA concentrations were significantly increased in the PL (Table 4) as a result of both doses as compared to control. BCAA increased (P<0.05) from 48–192 hrs and 24–120 hrs following the administration of 1.5 and 4.5 mg/kg DOX compared to control, respectively (Fig 2B). Ser increased (P<0.05) whereas Cys decreased (P<0.05) following the 1.5 mg/kg dose. Significant increases in Ser, Pro, Val, Lys, Iso and Leu were observed following the 4.5 mg/kg dose compared to control. His, ala, Pro, Val, Lys, Iso and Leu concentrations were more elevated (P<0.05) in the 1.5 mg/kg compared to 4.5 mg/kg treatment groups however Cys was significantly more elevated in the 4.5 mg/kg dose group. Ammonia was unaffected throughout the experiment.

**Table 4:** Intracellular amino acids in the plantaris.

Amino Acids	Dose	Time post-injection (in hours)								
	(mg/kg)	ctrl	24	48	72	96	120	144	168	192
Asp	1.5	0.32±0.03	0.41±0.08	0.79±0.1*	1.01±0.06*	1.17±.0.12*	1.15±0.08*	0.99±0.1*	0.95±0.07*	1.03±0.13*
	4.5	0.32±0.02	0.81±0.19	1.29±0.19*	1.07±0.09*	1.31±0.12*	0.95±0.05*	0.78±0.08	0.6±0.15	1.1±0.13*
Ser	1.5	0.89±0.09	0.99±0.22	1.65±0.39	2.37±0.38*	1.80±0.31	3.24±0.42*†	2.53±0.49*†	2.32±0.28	3.02±0.43*
	4.5	0.89±0.09	2.44±0.61*	2.7±0.44*	2.64±0.14*	2.0±0.16	1.29±0.11†	1.29±0.2†	1.46±0.39	2.73±0.25*
Glu	1.5	0.05±0.01	0.32±0.08*	0.31±0.04*†	0.65±0.1*	0.49±0.03*†	0.78±0.03*†	0.59±0.04*†	0.57±0.03*	0.65±0.08*†
	4.5	0.05±0.01	0.61±0.18*	0.61±0.09*†	0.57±0.05*	0.78±0.09*†	1.05±0.04*†	1.1±0.04*†	0.62±0.16*	1.7±0.2*†
Gly	1.5	0.27±0.01	0.27±0.05†	0.5±0.07	0.70±0.07*	0.83±0.09*	0.74±0.04*†	0.63±0.05*	0.62±0.03*	0.62±0.09*
	4.5	0.27±0.01	0.71±0.15*†	0.78±0.14*	0.71±0.08*	0.87±0.06*	0.6±0.05†	0.52±0.04	0.55±0.18	0.91±0.12*
His	1.5	0.45±0.02	0.7±0.15	1.58±0.22*	2.06±0.2*	2.31±0.25*	2.95±0.19*	2.19±0.15*	1.89±0.16*†	2.21±0.26*†
	4.5	0.45±0.02	2.0±0.53	4.83±1.83*	2.38±0.21	2.81±0.27	2.51±0.19	2.18±0.13	1.16±0.22†	3.79±0.43*†
Thr	1.5	0.5±0.03	1.04±0.6	1.31±0.1*	1.83±0.2*	1.65±0.11*	2.53±0.2*	1.93±0.13*	1.88±0.1*	2.0±0.22*
	4.5	0.5±0.03	2.12±0.52*	2.35±0.45*	2.09±0.17*	2.04±0.16*	2.56±0.23*	2.15±0.12*	1.67±0.45	2.19±0.15*
Ala	1.5	0.96±0.05	1.18±0.26	1.99±0.11*†	2.49±0.17*	2.73±0.27*	2.06±0.17*	1.83±0.23*	1.89±0.16*	2.09±0.2*
	4.5	0.96±0.05	2.55±0.53*	3.34±0.53*†	2.88±0.3*	2.22±0.16	1.76±0.09	1.43±0.11	1.24±0.42	2.1±0.29
Pro	1.5	0.11±0.01	0.12±0.02	0.18±0.02†	0.22±0.02*	0.26±0.03*	0.18±0.01	0.21±0.03*	0.21±0.02*†	0.22±0.03*
	4.5	0.11±0.01	0.21±0.05	0.37±0.07*†	0.3±0.04*	0.27±0.03*	0.22±0.02	0.15±0.01	0.1±0.03†	0.17±0.02
Cys	1.5	0.41±0.01	0.03±0.01*†	n/d	n/d	n/d	n/d	n/d	n/d	n/d
	4.5	0.41±0.01	1.13±0.29*†	3.6±0.33*	0.38±0.04	n/d	n/d	n/d	n/d	n/d
Tyr	1.5	0.03±0.002	n/d	0.06±0.01*†	0.10±0.01*	0.11±0.01*	0.09±0.01*†	0.08±0.01*	0.08±0.01*†	0.09±0.01*
	4.5	0.03±0.002	n/d	0.15±0.02*†	0.09±0.01*	0.13±0.02*	0.12±0.004*†	0.07±0.01*	0.05±0.01†	0.12±0.01*
Val	1.5	0.13±0.01	0.13±0.03	0.28±0.03*†	0.34±0.03*	0.32±0.03*	0.29±0.03*	0.32±0.03*†	0.3±0.03*†	0.29±0.03*
	4.5	0.13±0.01	0.26±0.06	0.5±0.09*†	0.35±0.03*	0.36±0.02*	0.3±0.02*	0.22±0.01†	0.14±0.03†	0.23±0.03
Met	1.5	0.003±0.002	0.05±0.01	0.07±0.01*†	0.1±0.01*	0.1±0.01*†	0.08±0.01*†	0.08±0.01*	0.08±0.01*†	0.08±0.01*
	4.5	0.003±0.002	0.97±0.03*	0.16±0.02*†	0.12±0.01*	0.13±0.01*†	0.11±0.01*†	0.07±0.01	0.05±0.01†	0.07±0.01
Lys	1.5	0.04±0.03	0.42±0.09	1.4±0.16*	1.38±0.21*	1.06±0.1*	1.43±0.19*†	1.52±0.16*†	1.51±0.03*†	1.23±0.14*
	4.5	0.04±0.03	0.97±0.25	1.75±0.24*	2.05±0.23*	1.1±0.04*	0.94±0.12†	0.87±0.12†	0.47±0.12†	1.19±0.14*
Iso	1.5	0.07±0.01	0.07±0.01	0.15±0.01*†	0.19±0.02*	0.17±0.02*	0.15±0.01*	0.17±0.02*†	0.16±0.01*†	0.16±0.02*
	4.5	0.07±0.02	0.14±0.03	0.29±0.05*†	0.2±0.02*	0.2±0.02*	0.16±0.01	0.12±0.01†	0.08±0.02†	0.13±0.02
Leu	1.5	0.19±0.01	0.15±0.03	0.29±0.03*†	0.38±0.03*	0.33±0.03*	0.3±0.02*	0.32±0.03*†	0.29±0.01*†	0.31±0.03*
	4.5	0.19±0.01	0.31±0.08	0.56±0.09*†	0.36±0.04*	0.38±0.04*	0.28±0.02	0.2±0.02†	0.13±0.03†	0.22±0.03
NH_3_	1.5	1.12±0.08	0.33±0.02	0.81±0.22†	1.09±0.16	1.66±0.52	0.96±0.11†	0.57±0.05	0.79±0.06	0.53±0.03†
	4.5	1.12±0.08	1.33±0.33	1.82±0.53†	1.26±0.06	0.91±0.09	0.51±0.013†	0.56±0.03	0.8±0.19	1.07±0.02†
EAA	1.5	2.9±0.3	6.6±1.4†	12.5±0.8*†	16.5±1.4*	15.9±1.1*	19.8±1.0*	15.2±1.4*	14.1±0.6*†	15.7±1.3*†
	4.5	2.9±0.3	17.6±4.2*†	19.2±1.6*†	19.0±1.3*	18.2±2.8*	20.0±1.4*	17.3±0.7*	8.2±1.0†	21.7±1.6*†

All values are means ± SEM. Concentrations represented in mmol/kg dry weight.

* denotes significance compared to control.

† Denotes significance between doses. n/d denotes not detected.

**Fig 3:**
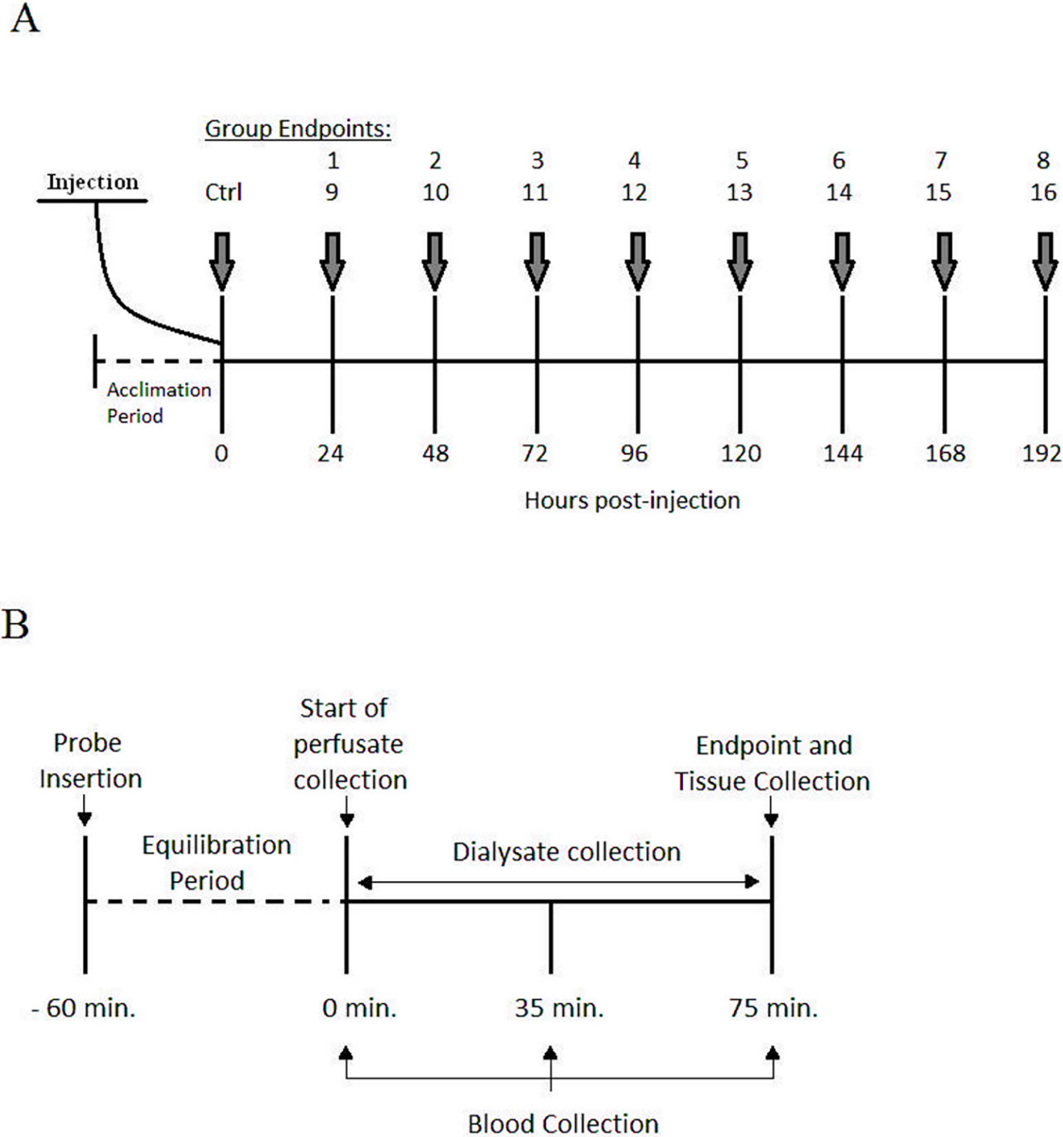
Schematic representation of the experimental groups and experimental procedures. (A) Representation of the experimental groups receiving an injection of 1.5 mg/kg dose (groups 1–8) or 4.5 mg/kg (groups 9–16) of DOX administered i.p. A sham injection is administered to the control group (Ctrl). (B) Representation of the experimental proceedings for dialysate, blood and tissue collection following microdialysis probe insertion and carotid artery cannulation.

SOL (Table 5) TAA (Fig 3E) and EAA concentrations were significantly increased as a result of both administered doses of DOX. Intramuscular BCAA concentrations where increased (P<0.05) after 48 hrs following the administrations of the 1.5 m/kg dose compared to control and remained elevated for the remainder of the experiments. BCAA were increased (P<0.05) 24–144 hrs following the administration of 4.5 mg/kg DOX compared to control (Fig 2C). The 4.5 mgs/kg dose of DOX resulted in a transient increase (P<0.05) in Asp, Gly, His, Lys, Iso and Leu compared to control and subsequently returned to baseline. Asp, Gly, His, Lys, Iso and Leu concentrations were higher following the 1.5 mg/kg compared to the 4.5 mg/kg dose of DOX. No significant changes in concentrations of ammonia were observed.

**Table 5:** Intracellular Amino Acids in the soleus.

Amino Acids	Dose	Time post-injection (in hours)								
	(mg/kg)	ctrl	24	48	72	96	120	144	168	192
Asp	1.5	0.18±0.02	0.21±0.02†	0.79±0.08*	0.89±0.13*	0.99±0.01*	0.63±0.02*	0.84±0.07*†	0.59±0.01*†	0.61±0.07*
	4.5	0.18±0.02	0.59±0.05*†	0.6±0.05*	0.6±0.06*	0.84±0.11*	0.87±0.17*	0.64±0.04*†	0.29±0.01†	0.5±0.09*
Ser	1.5	0.43±0.04	0.37±0.05†	1.4±0.3*	1.25±0.13*†	0.93±0.1	0.68±0.03	0.97±0.13†	0.74±0.12†	1.31±0.26*
	4.5	0.43±0.04	1.28±0.24*†	0.93±0.16	0.8±0.11†	1.12±0.31*	0.55±0.14	0.47±0.04†	0.4±0.07†	0.81±0.08
Glu	1.5	0.02±0.003	0.13±0.02†	0.32±0.02*†	0.48±0.03*†	0.39±0.05*	0.49±0.03*†	0.52±0.03*	0.34±0.03*	0.4±0.02*
	4.5	0.02±0.003	0.3±0.01†	0.25±0.02†	0.35±0.04*†	0.43±0.01*	1.05±0.15*†	0.58±0.04*	0.25±0.02	0.61±0.*
Gly	1.5	0.39±0.02	0.28±0.05†	1.17±0.14*	1.53±0.17*†	1.65±0.1*†	1.20±0.09*	1.56±0.04*†	1.05±0.1*†	1.15±0.13*
	4.5	0.39±0.02	0.94±0.12*†	0.94±0.1*	1.1±0.09*†	1.12±0.08*†	1.42±0.17*	0.88±0.06†	0.53±0.09†	1.23±0.23*
His	1.5	0.24±0.02	0.37±0.04†	1.79±0.28*	1.72±0.15*†	1.7±0.14*	1.45±0.03*†	1.89±0.18*	1.17±0.09*†	1.41±0.14*
	4.5	0.24±0.02	1.23±0.06†	1.93±0.53*	1.26±0.12*†	1.82±0.23*	2.23±0.35*†	1.68±0.14*	0.76±0.09†	1.7±0.09*
Thr	1.5	0.83±0.03	0.86±0.18†	3.64±0.09*	4.39±0.29*	3.4±0.18*	4.89±0.44*†	4.59±0.21*	3.77±0.25*†	4.51±0.3*
	4.5	0.83±0.03	3.06±0.58*†	4.09±0.55*	4.26±0.25*	3.55±0.32*	7.25±0.57*†	4.61±0.25*	2.71±0.2*†	4.34±0.11*
Ala	1.5	0.72±0.05	0.85±0.11†	2.16±0.29*	2.32±0.41*	2.48±0.19*†	1.15±0.06†	1.79±0.15*†	1.08±0.16†	1.42±0.11
	4.5	0.72±0.05	2.12±0.16*†	1.91±0.2*	1.8±0.28*	1.66±0.26*†	1.75±0.19*†	1.25±0.15†	0.61±0.11†	1.27±0.16
Pro	1.5	0.1±0.01	0.07±0.01†	0.23±0.03*	0.27±0.03*	0.27±0.01*	0.18±0.01†	0.28±0.02*†	0.21±0.01*†	0.22±0.03*†
	4.5	0.1±0.01	0.15±0.01†	0.21±0.02*	0.24±0.02*	0.23±0.03*	0.26±0.03*†	0.18±0.01†	0.08±0.004†	0.13±0.02†
Cys	1.5	0.48±0.03	0.2±0.001*†	n/d	n/d	n/d	n/d	n/d	n/d	n/d
	4.5	0.48±0.03	1.12±0.12†	1.91±0.36*	0.46±0.07	n/d	n/d	n/d	n/d	n/d
Tyr	1.5	0.03±0.002	n/d	0.08±0.01*†	0.11±0.01*†	0.11±0.01*	0.09±0.004*†	0.11±0.01*	0.07±0.004*†	0.07±0.01*†
	4.5	0.03±0.002	n/d	0.16±0.01*†	0.08±0.01*†	0.1±0.01*	0.16±0.01*†	0.1±0.01*	0.05±0.003†	0.1±0.01*†
Val	1.5	0.11±0.01	0.09±0.01†	0.36±0.04*	0.4±0.02*†	0.33±0.05*	0.27±0.01*†	0.42±0.03*†	0.27±0.01*†	0.24±0.03*
	4.5	0.11±0.01	0.24±0.02*†	0.37±0.05*	0.29±0.03*†	0.3±0.05*	0.37±0.04*†	0.28±0.03*†	0.15±0.02†	0.2±0.02
Met	1.5	0.03±0.003	0.04±0.004†	0.09±0.01*†	0.11±0.01*	0.11±0.01*	0.08±0.01*†	0.11±0.01*	0.07±0.003*†	0.07±0.01*†
	4.5	0.03±0.003	0.09±0.01*†	0.16±0.01*†	0.1±0.01*	0.12±0.01*	0.14±0.01*†	0.1±0.01*	0.05±0.01†	0.05±0.004†
Lys	1.5	0.39±0.07	0.22±0.02†	1.53±0.14*†	1.59±0.1*	0.97±0.15*	1.01±0.07*	1.55±0.07*†	1.19±0.09*†	0.86±0.05*†
	4.5	0.39±0.07	0.73±0.09†	0.89±0.07*†	1.71±0.17*	0.69±0.1	1.03±0.17*	0.89±0.14*†	0.39±0.03†	0.7±0.03†
Iso	1.5	0.06±0.01	0.04±0.004†	0.19±0.02*	0.21±0.01*†	0.17±0.03*	0.14±0.01*†	0.23±0.02*†	0.14±0.003*†	0.15±0.01*†
	4.5	0.06±0.01	0.12±0.01†	0.22±0.03*	0.16±0.02*†	0.16±0.03*	0.2±0.02*†	0.15±0.02*†	0.08±0.01†	0.1±0.01†
Leu	1.5	0.15±0.01	0.09±0.01†	0.34±0.03*	0.39±0.02*†	0.32±0.05*	0.25±0.01*	0.41±0.03*†	0.25±0.01*†	0.26±0.02*†
	4.5	0.15±0.01	0.27±0.03*†	0.39±0.05*	0.26±0.02†	0.27±0.04	0.31±0.03*	0.25±0.03†	0.13±0.01†	0.17±0.02†
NH_3_	1.5	1.38±0.12	0.36±0.06*†	0.92±0.04*†	1.54±0.15	1.05±0.09	1.06±0.04	1.3±0.1	0.67±0.05*†	0.66±0.04*†
	4.5	1.38±0.12	1.69±0.29†	1.94±0.21†	1.57±0.14	1.05±0.11	1.13±0.17	1.14±0.09	1.02±0.11†	1.65±0.08†
EAA	1.5	3.4±0.1	5.1±0.7†	18.8±0.4*	21.1±0.6*	17.9±1.1*	19.1±0.7*†	20.7±0.5*	15.2±0.4*†	17.6±0.6*
	4.5	3.4±0.1	14.2±3.1*†	18.1±2.0*	18.5±1.1*	18.3±1.4*	29.8±2.8*†	21.0±0.7*	9.4±0.8†	18.9±0.7*

All values are means ± SEM. Concentrations represented in mmol/kg dry weight.

* denotes significance compared to control.

† Denotes significance between doses. n/d denotes not detected.

## Discussion

The purpose of the current study was to examine the effects of DOX administration on amino acid concentrations in skeletal muscle, the interstitial space and the vasculature. The major findings were an observed increase in intramuscular TAA, EAA and BCAA concentrations, strongly suggesting that the administration of DOX resulted in a net increase in protein degradation. In addition, a dose-related difference was observed in the circulating concentration of amino acids where a greater increase was established following the administration of the 1.5 mg/kg dose of DOX compared to the 4.5 mg/kg dose. The current study provides novel insights into the response of amino acids in skeletal muscle and their subsequent concentrations in communicating compartments through the insterstitial space and into the circulation following the administration of DOX.

The net change in amino acids in the tissue is indicative of the balance between synthesis or degradation state of proteins in the muscle. The persistent three-fold increase in total intramuscular amino acids observed in the current study as a result of either administered dose of DOX strongly suggests that the rate of protein degradation was greater than that of protein synthesis. This is further substantiated by a similar increase in intramuscular EAA concentrations as a result of either dose of drug administered. *De novo* synthesis of EAA in the muscle is not possible and changes in intramuscular concentrations must come from the exchange with the vasculature or from the endogenous breakdown of protein within the tissue. Of the EAA, the branched-chain amino acids (BCAA) Val, Iso and Leu are the dominant essential amino acids oxidized in skeletal muscle. Therefore, any change in BCAA concentrations will most likely indicate a change in overall protein balance within the tissue.

The metabolic effects of BCAAs in skeletal muscle, which range from nitrogen homeostasis to energy production and protein synthesis, are well described [20–22]. In the current study, both the 1.5 and 4.5 mg/kg doses of DOX resulted in an increase in the intramuscular BCAA concentrations. The mitochondrial branched-chain amino acid aminotransferase (BCAT) isoenzyme is primarily repsonbile for initiating BCAA catabolism [23]. The activity of the BCAT which transfers an amino group from BCAA to α-ketoglutarate to form branched-chain ketoacids (BCKA) and Glu, responds rapidly to increases in intramuscular BCAA concentrations [24]. The increase in Glu synthesis is indicative of BCAT activity and formation of BCKAs. BCKA undergo irreversible oxidative decarboxylation by activated BCKA dehydrogenase complex (BCKAD) completing the the metabolism of leu (acetoacetate, acetyl-CoA), iso (propionyl-CoA, acetyl-CoA) and val (succinyl-CoA) for use in the TCA cycle [21].

The administration of 1.5 mg/kg DOX resulted in a substantial increase in BCAA in each muscle group whereas a transient increase was observed following the 4.5 mg/kg dose depending on the muscle group. The underlying mecahnisms responsible for the dose-related effect observed remains unclear. However, there is strong evidence to suggest that the increase in intramuscular BCAA is not due to a reduction in the rate of transamination by BCAT, which is not regulated by end-product inhibition or phosphorylation-dependent activation of inactivation mechanisms. This is made evident by the increase in Glu and Ala synthesis observed throughout the study.

In skeletal muscle, Glu is the key metabolic precursor for the synthesis of Ala and Gln via Ala aminotransferase and Gln synthase, respectively [25]. Ammonia detoxification in the muscle involves the safe transport of amino nitrogen, by Ala and Gln, to the liver for conversion to urea and subsequent excretion through the kidneys. Ala was increased in both the muscle and in the circulation which is consistent with previous research demonstrating the oxidation and release of Ala with enhanced supply of BCAA [26]. Due to analytical limitations, Gln concentrations could not be determined, however no consistent changes in circulating or intracellular ammonia were observed irrespective of the administered dose suggesting that the ammonia load was sufficiently managed via the increase in other disposal patways thus maitaining nitrogen homeostasis.

The anabolic effect of BCAAs on protein metabolism is well established. The mamalian target of rapamycin (mTOR) is a highly conserved serine-threonien kinase comprised of two disctinct mutiprotein complexes mTORC1 and mTORC2 [27, 28]. The mTORC1 complex includes mTOR, Raptor, Deptor, PRAS40 and mLST8/GβL. Intramuscular amino acid availability increases levels of Ca^2+^ which activates mTORC1 signaling via ca^2+^/calmodulin-mediate actiavtion of phosphoinositide 3-kinase (PI3K) [29, 30]. The potent BCAA-mediated stimulation of protein synthesis has been primarily attributed to the Leu regulated activation of mTORC1 by inducing the interaction of Ras-related GTP-binding protein (Rag) with ras-homolog enriched in the brain (Rheb) [31, 32]. Recently, it has been reported that DOX causes insulin resistance mediated by AMP-activated protein kinase (AMPk) inhibition [33]. Additionally, it has been shown that Leu activation of mTOR is partially dependant on insulin [32]. Taken together, it is possible that mTOR activity is stunted by the sum of these effects resulting in a decrease in protein synthesis.

Collectively, the current study shows that the administration of DOX did not affect BCAA availability and has increased intramuscular BCAA concentrations. The subsequent increases in Glu and Ala indicate that the enzymatic metabolism of BCAA has not been affected. Furthermore, it is clear that the possible redution in mTOR activity does not fully account for the increase in TAA observed in this study and that proteolysis may play a more prominent role.

Since its first clinical trials, DOX has been lauded as a highly effective anti-cancer chemotherapeutic. The fundamental concern with DOX chemotherapy, as is with most chemotherapeutic agents, is the systemic distribution of the drug following its administration. Previous research from our laboratory has demonstrated that DOX and DOXol is sequestered and retained in skeletal muscle under the same experimetal conditions as those used in the current study [17]. Once in the cell, DOX intercalates between DNA base pairs effectively disrupting cell replication. [12, 14]. It is possible that the integration of the drug in DNA may equally cause disruption in the unfolding of DNA and utimately disturbing or inhibiting protein synthesis and significantly contributing to the increase in intramuscular amino acid concentrations. The effect of DOX on regulatory mechanisms responsible for the proper formation of proteins has been studied. Recent research by Narandrula *et al*. [34] have shown that DOX capably induced rRNA distribution in cultured cells, suggesting that the formation of reactive oxygen species (ROS) and production of tumor necrosis factor alpha (TNFα) are likely factors responsible for the degradation of rRNA. Additionally, White and colleagues [35] have reported that DOX affects the phosphorylation of elongation factor 2 (EF-2), a key enzyme in the modulation of protein elongation during synthesis, and capably affects the rate of translation [36]. Furthermore, Lu *et al*. [37] have reported that DOX inhibits glucose-regulated protein 78 (GRP78) leading to the inactivation of unfolded protein response (UPR) and subsequent proteolysis via protease activity. The proteolytic outcome of protease activation may be the primary factor responsible for the significant increase in intramuscular TAA observed in this study.

Overall, the present study demonstrates that the administration of DOX increased intramuscular concentrations of BCAA which is not attributed to a reduction in BCAT activity and that the effect of the drug on BCAA-mediated protein synthesis alone does not account for the significant increase in intramuscular TAA. Seemingly, the disruption of protein synthesis by direct and indirect effects of DOX influences the dynamic balance between protein degradation and synthesis to favor the former over the latter. The resulting net protein breakdown is supported by the three-fold increase in intramuscular TAA and EAA concentrations.

Plasma concentrations of amino acids are a balance between production and elimination. Without ingestion, the largest shift in plasma amino acid concentrations come from changes in skeletal muscle protein turnover. As discussed above, the rate of protein degradation was greater than that of protein sythesis resulting in an increase of intramuscular TAA following the administration of the drug. The significant increase plasma TAA, where greater concentrations were observed following the 1.5 mg/kg dose compared to the 4.5 mg/kg dose of DOX, strongly indicates that the efflux of amino acid concentrations from the intramuscular pool into circulation were greater than that of systemic uptake resulting in the observed increase of arterial TAA. This is of particular interest as tumor cells experience abnormal growth and rapid division. To maintain the increase in biosynthesis and proliferation, cells increase amino acid uptake to meet metabolic demand [38, 39]. The increase in the availability of systemic amino acids may benefit tumor progression as tumorigenesis is optimized for a nutrient-poor microenvironment.

To maintain the increase in biosynthesis and proliferation, cells increase amino acid uptake to meet metabolic demand [38, 39]. The increase in the availability of systemic amino acids may benefit tumor progression as tumorigenesis is optimized for a nutrient-poor microenvironment.

The diffusion of amino acids between the tissue and vascular compartment must pass through the interstitial space (ISS). The interstitial compartment plays a functional role in the regualtion and integration of various endogenously produced metabolic substances [40, 41]. The current data demonstrate, for the first time, the effect of chemotherapy on interstitial amino acid concentrations. Fabris and MacLean [17] recently measured and discussed the presence of DOX in the interstital space. Interestingly, in the present study, an inverse relationship was observed between doses and the ISS concentrations of TAA. For example the 1.5 mg/kg dose of DOX resulted in a significant increase of TAA in the ISS compared to control whereas the 4.5 mg/kg dose resulted in a sustained decrease when compared to control. The increase in TAA concentrations followed by the eventual decline back to baseline is consistent with the rapid efflux of TAA out of the tissue and into the interstital compartment followed by a gradual uptake from the vasculature. The decrease of TAA in the ISS following the 4.5 mg/kg dose may be the result of secondary effects of the drug on trasporter proteins responsible for the flux of amino acids out of the tissue and into the ISS or from the ISS into the vasculature. The mediation of amino acid uptake and efflux from the myocyte is regulated by the expression of the several transporter proteins encoded by the solute carrier (SLC) superfamily [42, 43]. The difference between the 1.5 and 4.5 mg/kg dose may be the result of higher concentrations of DOX affecting the normal function of the transporter proteins. This is supported by our finding that lower concentrations of TAA were observed in the ISS and in the plasma as a result of the 4.5 mg/kg dose compared to the 1.5 mg/kg dose. No studies have been conducted that have shown that an increase in DOX interferes with the normal function of the various transporter proteins associated with amino acid transport in skeletal muscle. Additionally, the systemic effect of the 4.5 mg/kg dose on other tissues would help explain the lower increase of TAA in the plasma when compared to the 1.5 mg/kg dose. Therefore, in addition to a dose-related increase of plasma TAA, the study of the interstitial space by way of microdialysis has revealed a possible dose-related effect of DOX on amino acid transport mechanisms from skeletal muscle compartment into the vasculature.

This study describes for the first time the concurrent concentrations of amino acids in skeletal muscle, the interstitial space and the vascular compartment following the administration of an anti-cancer chemotherapeutic. Traditionally, the effects of DOX chemotherapy have been examined extensively on the heart and tumor with very little attention on skeletal muscle. The summation of restricted mTOR activity and upregulated proteolytic pathways are most likely responsible for the imbalance between protein synthesis and degradation, overwhelmingly favoring protein degradation. Depressed protein synthesis and loss of skeletal muscle mass this is a hallmark characteristic attributed to cachexia, a muscle wasting pathology and significant factor in the high mortality rate of cancer patients. This may have serious clinical implications where multiple doses of DOX chemotherapy administerered in a typical treatment regimen may accelerate the onset of cachexia and exacerbate the pathology. This study provides important insight into the possible mechanisms involved in the health and maintenance of skeletal muscle integrity following DOX chemotherapy and presents a substantial foundation for future studies focused on reducing skeletal muscle damage and recovery by targetting amino acid metabolism.

## Materials and methods

### Animals

The experimental procedures performed in this study were approved by the Laurentian University Animal Care Committee. Male Sprague-Dawley rats (n = 106, 434±60 gms) were obtained from Charles River Laboratories (Senneville, QC) and were housed and fed a 22/5 rodent diet (Envigo, WI, USA) according to Standard Operating Procedures and Policies for the Housing and Environmental Enrichment of Rodents at the Laurentian University Animal Care Facility. Experiments began following a one-week acclimation period.

All of the experiments and procedures in this study were approved by the Laurentian University Animal Care Committee. Male Sprague-Dawley rats (n = 106, 434±60 gms) were obtained from Charles River Laboratories (Senneville, QC) and were housed and fed a 22/5 rodent diet (Envigo, WI, USA) according to Standard Operating Procedures and Policies for the Housing and Environmental Enrichment of Rodents at the Laurentian University Animal Care Facility.

### Pre-experimental procedures

#### Experimental groups

The rats were anaesthetized with an EZ-150 vaporizer unit (EZ-Anesthesia, Euthanex Corporation, Palmer, PA). While under anaesthesia, the rats were injected intraperitoneally (i.p.) with Doxorubicin (Doxorubicin Hydrochloride. Pfizer, Canada) at a dose of 1.5 mg/kg (Groups 1 to 8) or 4.5 mg/kg (Groups 9 to 16). The administration of DOX followed an approved injections SOP for Laurentian University. A sham injection of saline solution was administered to a control group (i.p., n = 10). Once administered, rats were randomly grouped into experimental endpoints (n = 6) of 24, 48, 72, 96, 120, 144, 168 or 192 hrs. post-injection (Fig 3A).

#### Microdialysis probes

The construction of the microdialysis probes has previously been outlined [44]. Briefly, microdialysis fibers were constructed using Spectra Microdialysis fibers (Spectrum, VWR International) with a molecular cutoff of 13 kDa and Polyimide-100II tubing (MicroLumen, Tampa FL) cut to 9.5 and 5 cm lengths. The ends of the fiber were inserted 1 cm into the hollow polyimide tube and glued. The exposed portion of the fiber (diffusible) between the polyamide tubes measured 1.0 cm in length.

### Experimental procedures

#### Animals

On the day of the experiments, the rats were anaesthetized and remained under anaesthesia throughout the experiment. Heart rate and oxygen saturation were monitored throughout the experiment using a pulse oximeter (SurgiVet) attached to the base of the tail. Body temperature was maintained (between 36–37°C) using a heated surgical bed (EZ-Anaesthesia) and a heating lamp. The animals were euthanized following the completion of the experiment.

#### Microdialysis probe insertion

The skin of the leg was pulled back until the entire leg was exposed and the excess skin was then removed. A 21-gauge curved cannula was inserted anteriorly into the muscle along the muscle’s natural fibre orientation and acted as a guide for the microdialysis probe. Once the cannula was in place, the 9.5 cm portion of the probe is inserted caudally through the cannula and extended past the foot. Once the diffusible portion of the probe was oriented into the muscle, the 5 cm portion of the probe was held in place while the cannula was retracted out of the tissue leaving the probe in place.

#### Experimental protocol

Four microdialysis probes were inserted into a single hind limb, two in the lateral gastrocnemius and two in the medial gastrocnemius muscle (n = 4 probes per animal). Following probe insertion, the fibers were perfused (model 102, CMA) with Ringer’s solution at a rate of 3 μL/min. It is recognized that probe insertion results in some cellular disruption and therefore a 60 min. equilibration period prior to the initiation of the experiment was used to insure that the external environment surrounding the probes had stabilized and all cellular damage had dissipated [45]. Subsequently, the skin and tissue covering the left carotid artery was removed. A 21-gauge cannula was inserted into the exposed artery and sutured in place. Heparinized saline (100 μL) was injected into the artery to maintain patency in preparation for blood sampling.

Following the period of equilibration, the experimental protocol was initiated (Fig 3B) where dialysate was collected for a period of 75 min in order to obtain sufficient volume that was required for analysis. Dialysate samples were collected in microcentrifuge tubes and immediately sealed to prevent evaporation and stored at -80°C until analysis. Arterial blood samples were collected during the collection of dialysate at 0, 35 and 75 min. Samples were spun at 15,000 rpm for 25 sec., plasma was separated and stored at -80°C until analysis. Upon completion of dialysate and blood collection the medial gastrocnemius (WG), soleus (SOL) and plantaris (PL), soleus (SOL) muscles from the contralateral leg used for microdialysis were excised, inserted into cryogenic vials, flash frozen in liquid nitrogen and stored at -80°C until analysis.

### Sample analysis

#### Skeletal muscle

Flash frozen muscle samples were placed in a freeze drier (Freeze Drier 4.5, Labconco) at 10 microns Hg for 24 hrs. Samples were then powdered and tweezed free of all visible connective tissue. Two mg of freeze dried muscle sample were added to 100 μL purified water (EMD Millipore, USA) containing L-Norleucine (Sigma-Aldrich, USA) as internal standard then homogenized for 30 sec. using a Kontes Pellet Grinder. The homogenate was transferred into a centrifugal filtering tube (EMD Millipore, USA) and centrifuged at 15 000 r.p.m. for 20 min. 20 μL of the filtrate was added to 60 μL borate buffer and 20 μL fluor reagent (AccQ-Tag, Waters Canada) and vortexed immediately for 30 sec. Samples were placed on a heating block at 55°C for 10 min. before analysis.

#### Plasma

Plasma samples were transferred into centrifugal filtering tubes (EMD Millipore, USA) and centrifuged at 15 000 r.p.m. for 20 min. 20 μL of the filtrate was added to 60 μL borate buffer and 20 μL fluor reagent (AccQ-Tag, Waters Canada) and vortexed immediately for 30 sec. Samples were placed on a heating block at 55°C for 10 min. before analysis.

#### Dialysate

The collected dialysate samples represent an ultra-clean filtrate and did not require any pre-analysis preparation. As such, 20 μL of dialysate was added directly to 60 μL borate buffer and 20 μL fluor reagent (AccQ-Tag, Waters Canada) and vortexed immediately for 30 sec. Samples were placed on a heating block at 55°C for 10 min. before analysis. Dialysate and perfusate blanks as well as dialysate samples from control animals were used to confirm the quality and purity of the samples analyzed.

#### Amino acid quantification

Amino Acid concentrations were determined by HPLC (Waters e2695, 2475 Fluorescence Detector, Waters Corporation) using AccQ-Tag Amino Acid Analysis kits (Waters, Canada). Derivatized standards include aspartate, serine, glutamate, glycine, histidine, ammonia, arginine, threonine, alanine, proline, cysteine, tyrosine, valine, methionine, lysine, isoleucine, leucine and phenylalanine. Quantification of amino acids were determined by comparison with a standard curve and skeletal muscle amino acids where corrected by internal standard.

#### Statistics

Changes in amino acid concentrations between time points and doses were analyzed using a two-way ANOVA. When significant differences were indicated, a Tukey’s *post hoc* test was used to determine where the significance occurred. Significance was accepted at P<0.05. All tabled values and values used in tables and graphical representations are displayed as mean ± SEM.
